# Simultaneous high-resolution scanning Bragg contrast and ptychographic imaging of a single solar cell nanowire

**DOI:** 10.1107/S1600576715017975

**Published:** 2015-11-10

**Authors:** Jesper Wallentin, Robin N. Wilke, Markus Osterhoff, Tim Salditt

**Affiliations:** a Institute for X-Ray Physics, University of Göttingen, Friedrich-Hund-Platz 1, Göttingen, 37077, Germany

**Keywords:** X-ray diffraction, nanowires, solar cells, ptychography

## Abstract

Simultaneous scanning Bragg contrast and small-angle ptychographic imaging of a single solar cell nanowire are demonstrated, using a nanofocused hard X-ray beam and two detectors. The 2.5 µm-long nanowire consists of a single-crystal InP core of 190 nm diameter, coated with amorphous SiO_2_ and polycrystalline indium tin oxide. The nanowire was selected and aligned in real space using the small-angle scattering of the 140 × 210 nm X-ray beam. The orientation of the nanowire, as observed in small-angle scattering, was used to find the correct rotation for the Bragg condition. After alignment in real space and rotation, high-resolution (50 nm step) raster scans were performed to simultaneously measure the distribution of small-angle scattering and Bragg diffraction in the nanowire. Ptychographic reconstruction of the coherent small-angle scattering was used to achieve sub-beam spatial resolution. The small-angle scattering images, which are sensitive to the shape and the electron density of all parts of the nanowire, showed a homogeneous profile along the nanowire axis except at the thicker head region. In contrast, the scanning Bragg diffraction microscopy, which probes only the single-crystal InP core, revealed bending and crystalline inhomogeneity. Both systematic and non-systematic real-space movement of the nanowire were observed as it was rotated, which would have been difficult to reveal only from the Bragg scattering. These results demonstrate the advantages of simultaneously collecting and analyzing the small-angle scattering in Bragg diffraction experiments.

## Abstract 2

Simultaneous Bragg diffraction and coherent small-angle scattering of a single solar cell nanowire is demonstrated, using a nanofocused hard X-ray beam and two detectors.

## 1. Introduction   

Semiconductor nanowires are crystalline nanostructures which are intensely researched in areas such as electronics (Tomioka *et al.*, 2012 ▸), light-emitting devices (Duan *et al.*, 2003 ▸) and quantum physics (Mourik *et al.*, 2012 ▸). In particular, nanowire-based solar cells have shown strong development in recent years (Wallentin *et al.*, 2013 ▸; Colombo *et al.*, 2009 ▸; Yao *et al.*, 2014 ▸; Goto *et al.*, 2009 ▸; Borgström *et al.*, 2011 ▸). An electrical connection must be made to the as-grown nanowires to complete the device, which is normally done by depositing noncrystalline thin films of insulators and conductors. Such films can induce strong strain in the semiconductor (Bouwes Bavinck *et al.*, 2012 ▸), which can affect for instance the electronic bandgap (Bouwes Bavinck *et al.*, 2012 ▸) and the free carrier mobility (Lee *et al.*, 2005 ▸).

Transmission electron microscopy can be used to investigate strain in bare nanowires (Larsson *et al.*, 2007 ▸), but the strong absorption of electrons makes this method challenging for processed nanowire devices. X-ray diffraction (XRD) has been a key method in the study of crystalline materials for a century, and the long penetration length of X-rays allows investigations of complete devices (Hrauda *et al.*, 2011 ▸). Owing to the difficulty in making efficient X-ray optics, XRD has traditionally been used to probe relatively large sample areas. With recent developments in high-brilliance synchrotron radiation sources and X-ray focusing optics, the real-space resolution has increased. It has become possible to probe many types of individual nanostructures (Hrauda *et al.*, 2011 ▸; Mocuta *et al.*, 2008 ▸; Williams *et al.*, 2003 ▸; Chamard *et al.*, 2008 ▸; Newton *et al.*, 2009 ▸; Hanke *et al.*, 2008 ▸; Robinson & Harder, 2009 ▸), which can differ substantially from the sample average (Mocuta *et al.*, 2008 ▸). Lattice changes due to strain (Mastropietro *et al.*, 2013 ▸; Newton *et al.*, 2009 ▸; Hrauda *et al.*, 2011 ▸), materials composition (Haag *et al.*, 2013 ▸; Hrauda *et al.*, 2011 ▸), heating (Haag *et al.*, 2013 ▸) and crystal polytypes (Favre-Nicolin *et al.*, 2010 ▸; Chamard *et al.*, 2008 ▸) have been investigated. For composite devices such as the one studied here, a limitation of Bragg scattering is that it does not give information about noncrystalline regions.

A key challenge with Bragg scattering of nanostructures is to align the sample and the beam in both real and reciprocal space (Stangl *et al.*, 2014 ▸; Robinson & Harder, 2009 ▸). The real-space alignment requires positioning and controllably moving the sample in the nanofocused beam, with a precision that should be significantly smaller than the focus size. The reciprocal-space requirement means that the beam and the sample lattice must be aligned at the correct angle to fulfil the Bragg condition. To collect rocking curves the sample is rotated in a small angular range around a Bragg peak. Since the sample can never be perfectly positioned at the center of rotation, this leads to a shift in real space. As the sample feature sizes and X-ray foci shrink far below 1 µm, the alignment problem becomes increasingly severe.

Alignment for small-angle (forward) X-ray scattering is easier, since the positioning only has to be carried out in real space. It is based on scattering from all electrons in the sample, without interference induced by crystal planes, and essentially measures the projected electron density. Small-angle scattering can therefore give complementary information to the Bragg scattering (Gallagher-Jones *et al.*, 2014 ▸; Bunk *et al.*, 2009 ▸). Here, we combine small-angle and Bragg scattering to investigate a solar cell nanowire with a nanofocused X-ray beam. Ptychographic reconstruction of the small-angle scattering was used to find and track the real-space position with sub-beam-size spatial resolution. While the small-angle scattering shows a quite homogeneous profile, the scanning Bragg diffraction microscopy reveals strong inhomogeneity in the crystalline InP core.

## 2. Experimental   

**Figure 1:**
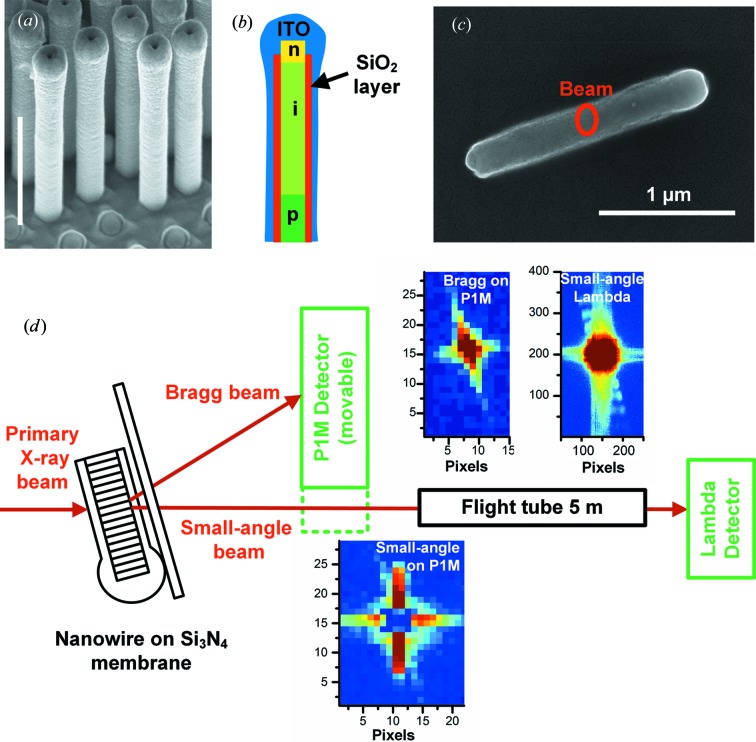
(*a*) Scanning electron microscopy image of nanowires in a solar cell, standing on the growth substrate. Scale bar 1 µm, tilt 45°. Stubs from broken off nanowires can be seen in the lower part. (*b*) Drawing depicting the crystalline nanowire with its p-i-n doping structure, the amorphous SiO_2_ film and the polycrystalline ITO layer. The thicknesses of the layers have been exaggerated for clarity. (*c*) Scanning electron microscopy image of the nanowire that was investigated for this paper, lying on the Si_3_N_4_ membrane, taken after the experiments. The red ring shows the size of the nanofocused X-ray beam. (*d*) Drawing showing the experimental setup, as seen from above. Two detectors were used, of which the Pilatus 1M closer to the sample could be moved in the horizontal direction, in and out of the small-angle beam. Examples of parts of detector images are shown, with the scale in pixels. The color scales have been adjusted to enhance the visibility of the streaks from the nanowire, which makes the high-intensity central regions appear oversaturated. In the small-angle detector images there are vertical and horizontal streaks from the KB mirrors.

The nanowire investigated here was taken from a solar cell, in which about four million nanowires in a dense matrix (0.47 µm pitch) make up a 1 × 1 mm device. The nanowire solar cell preparation has been described previously (Wallentin *et al.*, 2013 ▸; Borgström *et al.*, 2011 ▸). Briefly, gold seed particles were first defined by nanoimprint lithography on an InP substrate. Then, 190 nm-diameter InP nanowires doped with an axial p-i-n junction were grown to a length of about 2.5 µm, after which the gold seed particles were removed by wet etching. To create a top contact, an insulating SiO_2_ film was deposited by atomic layer deposition to prevent axial short-circuiting, and a sunlight-transparent top contact of indium tin oxide (ITO) was sputter deposited. The ITO sputtering gives an inhomogeneous coverage of the nanowire, with a thicker layer at the top (Figs. 1 ▸ *a* and 1 ▸ *b*). Thus, the nanowires consist of a single-crystal InP core, an amorphous SiO_2_ film and a polycrystalline ITO layer. Similar devices have shown an efficiency of 13.8% (Wallentin *et al.*, 2013 ▸), while this cell showed an efficiency of 9%.

**Figure 2:**
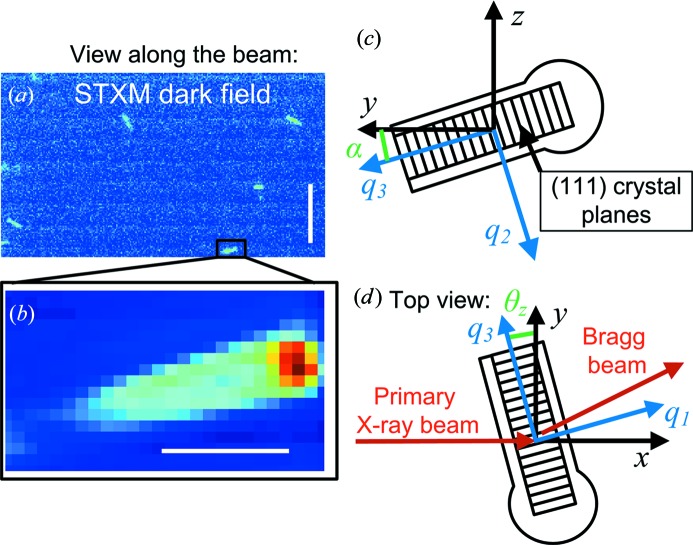
Rotational alignment. (*a*) Overview dark-field STXM image of the nanowire sample. Scale bar 10 µm, step size 200 nm. Several nanowires can be seen lying on the Si_3_N_4_ membrane. The horizontal stripes are due to variations in the synchrotron ring current. (*b*) High-resolution dark-field STXM image of a single nanowire from the boxed area in (*a*). Scale bar 1 µm, step size 100 nm. (*c*) Drawing depicting the nanowire as seen facing the beam. The (111) planes are oriented orthogonal to the long axis. Therefore, the scattering vector **q** is parallel to the nanowire long axis. The angle between the nanowire and the horizontal *y* axis is α = 16°. (*d*) Drawing depicting the nanowire as seen from above. The sample was rotated around the vertical *z* axis. The rotation around the vertical *z* axis, θ*_z_*, needed to meet the Bragg condition is larger than the Bragg angle, θ_B_, owing to the rotation α.

For the X-ray investigations, nanowires were scraped off the growth substrate with clean-room tissue and deposited randomly on an X-ray-transparent 1 µm-thick Si_3_N_4_ membrane (Fig. 1 ▸ *c*). The GINIX (Göttingen Instrument for Nano-Imaging with X-rays) end station at the P10 beamline at the PETRA III synchrotron, DESY, Hamburg, Germany, was used for the X-ray investigations (Fig. 1 ▸ *d*) (Salditt *et al.*, 2015 ▸). The beam of energy 13.8 keV was focused using Kirkpatrick–Baez (KB) mirrors to a size of (FWHM of the intensity) 140 × 210 nm in the horizontal and vertical directions, respectively, as determined by ptychographic reconstruction [see below and Wilke *et al.* (2014 ▸)]. The real-space coordinate system (*x*, *y*, *z*) is defined in Fig. 2 ▸. A sample stage optimized for tomography was used, rather than a diffractometer, with a single rotation around the vertical *z* axis (θ*_z_*) and high-precision piezo motors for translation.

Two different single-photon counting detectors were used, as shown in Fig. 1 ▸(*d*). Near the sample, at *x* = 0.38 m, there was a Dectris Pilatus 1M detector (P1M), with 172 µm pixel size, which could be moved in and out of the small-angle beam. There was also a new Lambda detector based on the Medipix 3 chip with 55 µm pixel size (Pennicard *et al.*, 2013 ▸; Wilke *et al.*, 2014 ▸) positioned at *x* = 5.1 m, behind a flight tube, which was used together with a semi-transparent central stop to improve the dynamic range (Wilke *et al.*, 2013 ▸).

## 3. Alignment   

Different strategies have been used to align nanocrystals for Bragg scattering experiments. X-ray fluorescence maps can be used for real-space alignment if the sample has a suitable absorption edge (Hanke *et al.*, 2008 ▸). If epitaxically grown nanocrystals are investigated on the growth substrate, the sample can first be aligned in reciprocal space, by using the strong substrate Bragg reflection (Mocuta *et al.*, 2008 ▸). After alignment in reciprocal space, the sample can then be scanned in real space until a single nanostructure is found. In many cases, such as the present one, the substrate and the nanocrystal are of the same material, which means that the nanocrystal signal would have to be detected against a much stronger substrate signal. Furthermore, in order to measure single nanowires in such a dense nanowire array, a diffraction angle close to the nanowire axis would have had to be used. The diffraction signal would then have been averaged over a large segment of the nanowire, degrading the spatial resolution with regards to the optimum size given by the X-ray beam focus.

Instead, we chose to measure single nanowires, which had been broken off from the growth substrate, and to start with the real-space alignment. This approach completely removes any scattering from the growth substrate and allowed using a low-index Bragg reflection. This method should preserve the nanowires in their original strain state, except very near the base, since the processing layers remain intact, but further experiments comparing standing and scraped off nanowires should be performed to verify this. After coarse alignment with an in-line optical microscope, we made a large-area scanning transmission X-ray microscopy (STXM) image with sufficient resolution to reveal individual nanowires (step size 200 nm; Fig. 2 ▸ *a*). Then, a single nanowire was chosen and imaged with a smaller STXM image, step size 100 nm, as shown in Fig. 2 ▸(*b*). In STXM, the sample is two-dimensional raster scanned in the beam, and detector images are acquired at each point. From these images, different contrast modes can be evaluated, as discussed in §[Sec sec4]4.

Since the rotational alignment could not be carried out using a substrate peak, the measured shape of the nanowires was used instead. InP nanowires grow in the cubic zincblende (111)B direction, with the (111) crystal planes oriented orthogonal to the long axis (Fig. 2 ▸ *c*) (Hiruma *et al.*, 1995 ▸). During crystal growth, a high density of wurtzite crystal segments are also formed, so the nanowire is a polytypic mixture. We were nominally measuring the Bragg reflection for the zincblende (111) planes, whose scattering vector is parallel with and only 0.36% larger than that of the wurtzite 000.2 reflection (Kriegner *et al.*, 2011 ▸). For simplicity, we refer only to the cubic 111 reflection. The 111 reflection has a Bragg angle of θ_B_ = 7.62° at X-ray energy 13.8 keV. The small angle, almost orthogonal to the nanowire axis, preserved the high spatial resolution of the nanofocused beam. The rotational alignment of the membrane was first done with the membrane parallel with the beam (*θ_z_* = 90°), using knife-edge scans.

The correct θ*_z_* for the Bragg condition is not θ_B_, since the nanowire was rotated in the plane of the sample membrane. However, the in-plane rotation α for the chosen nanowire could be readily evaluated from the STXM scans. Since the (111) planes are orthogonal to, and the scattering vector **q** is parallel with, the nanowire long axis, the rotation is (Appendix *A* [App appa]) 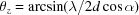. With the measured α = 16°, we calculated θ*_z_* = 8.0°. Rocking curve measurements were performed (see Fig. 6*a* in §5), giving a peak value of 10.88°. The deviation is presumably because the ITO film on the nanowire was thicker at the top, which meant that the nanowire axis was not perfectly aligned with the membrane. The geometrical calculation also predicts the vertical and horizontal deflection of the Bragg peak on the detector, which was (*y*, *z*) = (9.88, 3.07) cm compared with the measured value (9.89, 2.81) cm. Since the Bragg angle can be accurately predicted it would be possible to use a small high-resolution detector placed further away for increased angular resolution.

In Fig. 1 ▸(*d*), examples of the regions of interest (ROI) in detector images are shown. In both small-angle and Bragg scattering, the diffraction peaks are to first order a convolution of the Fourier transforms of the probe beam and the shape function of the object (Hruszkewycz *et al.*, 2012 ▸; Robinson *et al.*, 2001 ▸). The shape function is defined as 1 in the object and 0 outside of the object. For a rod-shaped object, the two-dimensional Fourier transform of the shape function generates streaks orthogonal to the long axis of the rod. Streaks orthogonal to the nanowire axis can therefore be seen in all three detector images. In the small-angle scattering image, vertical and horizontal streaks from the KB mirrors can also be observed.

## 4. Multi-mode imaging   

**Figure 3:**
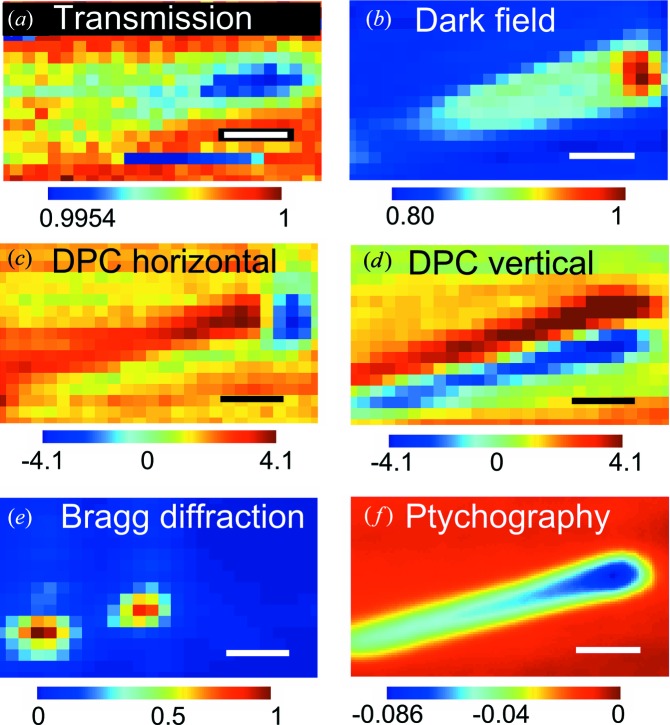
Multimode imaging analysis of a single scan. Step size 100 nm, scale bars 0.5 µm. (*a*) Transmission STXM, normalized intensity. The data were modified to compensate for systematic intensity fluctuations due to synchrotron ring top-ups. (*b*) Dark-field STXM image, normalized intensity. (*c*) Differential phase contrast in the horizontal direction, in 10^6^ rad m^−1^. (*d*) Differential phase contrast in the vertical direction, in 10^6^ rad m^−1^. (*e*) Scanning Bragg diffraction microscopy, normalized intensity. (*f*) Ptychographic reconstruction, phase shift in rad.

After alignment of the sample in both real and reciprocal space, STXM scans were performed with the P1M detecting the Bragg peak and the Lambda detector measuring the small-angle scattering (step length 100 nm, exposure time 1 s per point). Several imaging contrast modes can be calculated from the small-angle scattering (Fig. 3 ▸) (Thibault *et al.*, 2009 ▸; Bunk *et al.*, 2009 ▸), all of which probe both crystalline and noncrystalline parts of the sample. The transmission (bright-field) STXM is here defined as the total intensity in the small-angle scattering ROI, corresponding to the size of the propagated beam on the detector. This signal is modulated by absorption in the object. Conversely, dark-field STXM is here defined as the total signal outside of the same ROI. It is therefore a measure of the amount of scattered light. Differential phase contrast (DPC) is based on an evaluation of the center of mass (COM) of the scattered signal on the detector, which is modified by density gradients in the object.

While the contrast in transmission STXM is poor (Fig. 3 ▸ *a*), about 0.5%, the dark-field image shows about 20% contrast (Fig. 3 ▸ *b*). The DPC images show contrast at the nanowire edges (Figs. 3 ▸ *c* and 3 ▸ *d*), where there is a gradient in electron density. Since the nanowire was oriented almost horizontally, the shape of the nanowire is most easily seen in the vertical DPC.

The Bragg signal on the P1M detector was analyzed per spatial point to create a two-dimensional image (Mocuta *et al.*, 2008 ▸). We use the term scanning Bragg diffraction microscopy (SBM) for this method, to distinguish it from the scanning small-angle diffraction methods which have different contrast mechanisms (Thibault *et al.*, 2008 ▸). The SBM image in Fig. 3 ▸(*e*) was achieved by integrating the intensity of the Bragg peak ROI, per spatial point, similar to transmission STXM. The SBM image shows the spatial distribution of the Bragg scattering for a particular angle and only probes the single-crystal InP core. It shows major differences compared with the images from small-angle scattering, with two strongly scattering regions in the middle and lower end, but no signal from the top part of the nanowire. The spatial resolution in all these imaging modes is limited by the size of the focused beam, but coherent imaging techniques can reach sub-beam-size resolution by iteratively reconstructing the phase (Miao *et al.*, 1999 ▸).

A requirement for such reconstruction methods, however, is that the scattered beam is sufficiently oversampled (Miao *et al.*, 1998 ▸). In single-exposure coherent diffraction imaging, the oversampling ratio, σ, should be larger than two. For a one-dimensional object of size *L* = 200 nm, X-ray wavelength λ = 0.899 Å, detector distance *z*_D_ and pixel size *x*_D_, we have in the far-field geometry 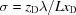. For the small-angle scattering at the Lambda detector, we get σ ≃ 42, while for the Bragg scattering at the P1M we find σ ≃ 1. We employed an alternative coherent imaging method, ptychography, where the object is scanned in the beam with an overlapping exposure between different positions (Rodenburg *et al.*, 2007 ▸). It is more complicated to define the sampling ratio for ptychography owing to the overlapping exposures, and the criterion could be considerably relaxed with this method (Edo *et al.*, 2013 ▸). The one-dimensional sampling ratio is still proportional to 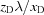, as in single-exposure CDI, which in our experiment means that it is very difficult to reconstruct the phase for the Bragg scattering. The available detectors and the geometry of the beamline did not allow better sampling of the Bragg scattering at the time of the experiment, but this is not a fundamental limitation of our method.

**Figure 4:**
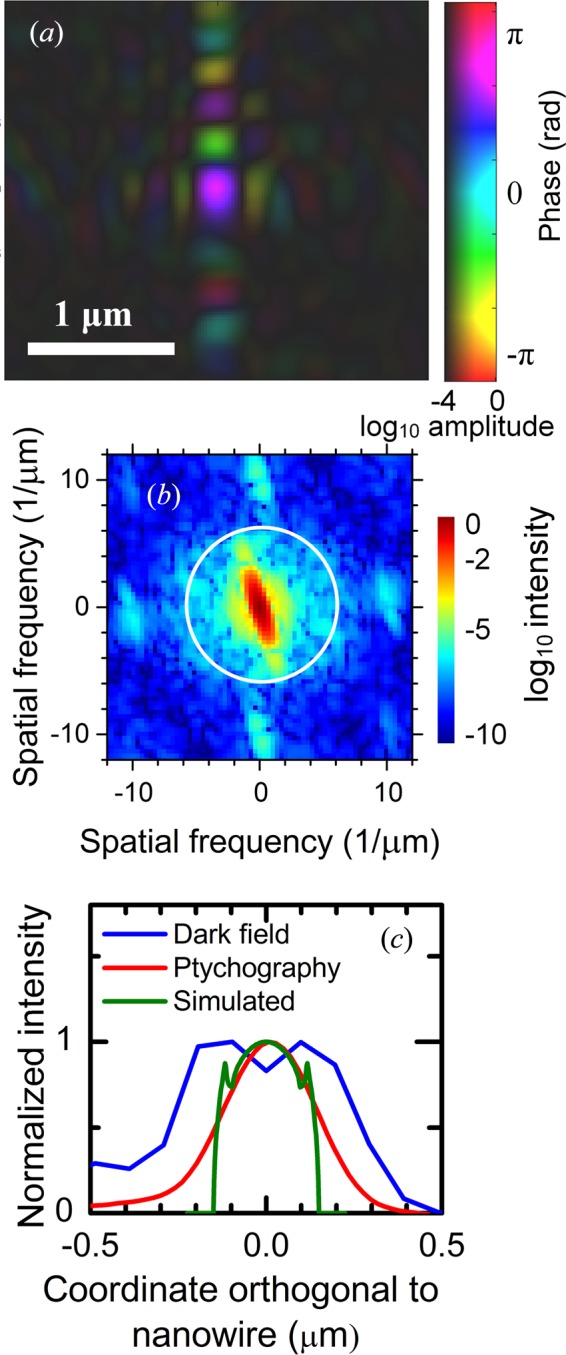
Ptychography. (*a*) Phase and amplitude of reconstructed probe beam. (*b*) Power spectral density of the reconstructed nanowire in Fig. 3 ▸(*f*). The white ring indicates 6 µm^−1^. (*c*) Cross-sectional profiles, averaged along a section of the nanowire, and the simulated phase shift. All profiles were normalized to their maximum.

The STXM data were used to make a ptychographic reconstruction of the small-angle scattering data with the ePIE algorithm (reconstruction parameters α = β = 0.5) (Maiden & Rodenburg, 2009 ▸; Wilke *et al.*, 2014 ▸), which simultaneously reconstructs the object and the probe beam (Figs. 3 ▸ *f* and 4*a*). The resulting image has higher resolution than the STXM modes, and the thicker head can be clearly seen. In the power spectral density map of the ptychographic image (Fig. 4 ▸ *b*) the contributions above the background extend to approximately *ν* = 6 µm^−1^, as indicated by a white ring, corresponding to a half-period real-space pixel size (1/2*ν*) of about 80 nm (Shapiro *et al.*, 2005 ▸; Wilke *et al.*, 2014 ▸).

To get a better comparison of the spatial resolutions of the different imaging modes, averaged line profiles were calculated across the nanowires (Fig. 4 ▸ *c*). The expected phase shift profile was also calculated, based on the nominal layer thicknesses and material data. The calculated profile is almost box shaped, with two 10 nm-thick peaks at the edges, since the InP core and the ITO film have the highest electron density and phase shift (δ = 4.6 × 10^−6^ and 6.9 × 10^−6^, respectively) and the SiO_2_ film has the lowest (δ = 2.9 × 10^−6^). The FWHM of the dark-field STXM was 0.54 µm, which is reasonable considering a convolution of a 0.31 µm object with a 0.21 µm beam. The profile of the ptychographic image showed a Gaussian profile with an FWHM of 0.30 µm. Although this is similar to the nanowire diameter, the profile should ideally not be Gaussian shaped. Thus, the spatial resolution was not sufficient to distinguish the different layers in the core–shell structure, but was good enough to resolve variations along the nanowire axis. The SBM scans showed lower-diameter cross sections than the STXM images, which is expected since they only probe the 0.19 µm-diameter crystalline core. While the SBM scans in Fig. 3 ▸ had too few data points to allow a meaningful analysis, a similar analysis of the higher-resolution SBM scans (see below) showed an FWHM of 0.44 µm. Ptychography is quantitative, in the sense that the reconstruction gives absolute values for the phase shift which can be used to calculate the projected electron density (Giewekemeyer *et al.*, 2010 ▸). The measured phase shift in the center of the body of the nanowire was −44 mrad, which is only half of the calculated −90 mrad. This discrepancy may be related to the limited resolution.

## 5. Spatial variations of the scattering vector   

**Figure 5:**
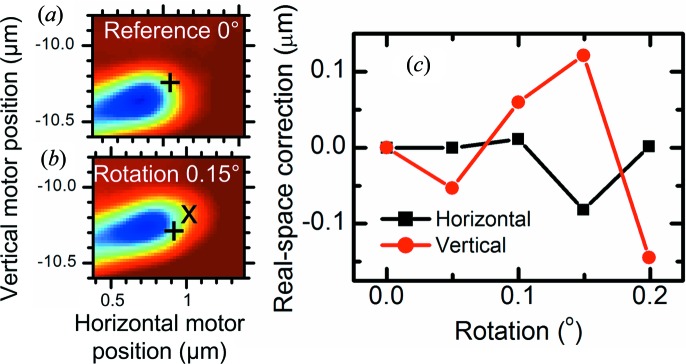
Measuring real-space movement due to rotation. (*a*) Parts of ptychographic reconstructions, such as in Fig. 3 ▸(*f*). The + sign indicates the reference point. In the direction across the nanowire, this point was calculated from the average of the intensity summed along the nanowire axis. In the direction along the nanowire axis, this point was the half-maximum of the slope at the tip. The horizontal coordinate is the motor position. The scan ranges were adapted to the systematic movement of 7.5 µm per degree. (*b*) Part of a ptychographic reconstruction, taken at a relative rotation around the *z* axis of +0.15°, compared with (*a*). Here, the × sign indicates the reference point calculated from this image. The + sign indicates the expected position of this reference point, calculated from the systematic shift in the **y** direction. (*c*) Remaining shift of the reference point *versus* relative rotation, after correction for systematic movement. This is the distance between the measured (‘×’) and expected (‘+’) positions of the reference points, as in (*b*).

Next, the multimode imaging was repeated at five different rotations. The object was not perfectly positioned at the center of rotation, so that the sample therefore systematically moved in the **y** direction during rotation. Using STXM scans we could characterize and compensate for this movement, which we measured to be 7.5 µm per degree. However, the ptychographic images revealed that there was residual nonsystematic real-space movement of the sample of the order of 100 nm per 0.05° step (Fig. 5 ▸), both horizontally and vertically. Note that such movement would be difficult to observe only from the Bragg scattering.

**Figure 6:**
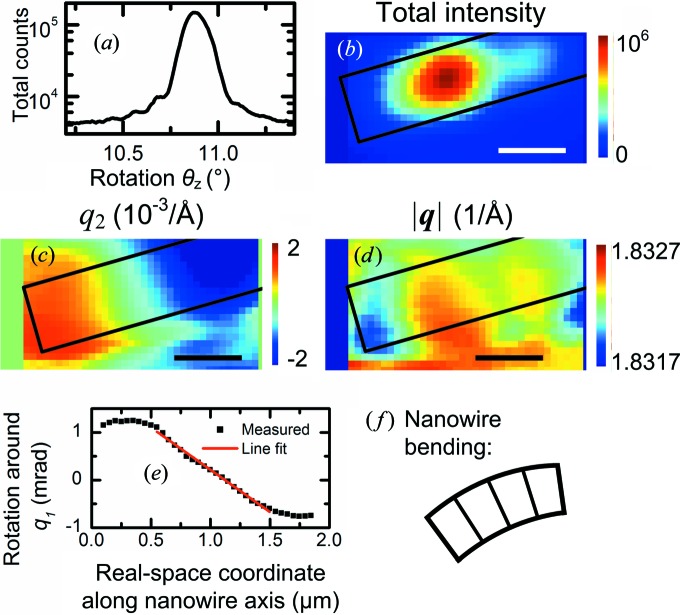
High-resolution scanning Bragg diffraction microscopy of the left (lower) half of the nanowire. (*a*) Rocking curve of θ*_z_*, around the *z* axis, from the highest-intensity region near the base of the nanowire. The FWHM is 0.14°. (*b*) Sum of the intensity in the Bragg peak ROI of all angles. (*c*), (*d*) Images of *q*_2_ and |**q**|, respectively. Each SBM scan was first shifted in order to compensate for real-space movement. The boxes in (*b*)–(*d*) indicate the position of the nanowire, as evaluated from the small-angle scattering. Scale bars are 0.5 µm, step size 50 nm. The position of the Bragg peak can be measured and analyzed also outside the boxed region, since the focused X-ray beam has intensity outside of the focus (see Fig. 4 ▸ *a*). (*e*) The rotation around *q*_1_, calculated as β = −*q*_2_/*q*_3_ and averaged across the nanowire, *versus* a real-space coordinate, *s*, along the nanowire axis (parallel with *q*_3_). The red line shows a linear fit with slope dβ/d*s* = −1.78 mrad µm^−1^. (*f*) Exaggerated sketch of how the nanowire bends in the plane of the membrane, as seen along the beam (compare with Fig. 2 ▸ *c*).

In Fig. 6 ▸(*a*), a rocking curve around the vertical *z* axis is shown for the point with highest Bragg intensity near the base of the nanowire, with a peak value of θ*_z_* = 10.88° and an FWHM of 0.14°. The lower half of the nanowire was investigated with high-resolution scans (50 nm step size) at five rotations (Fig. 6 ▸): 10.78, 10.83, 10.88, 10.93 and 10.98°. For each real-space point, the data were first shifted to compensate for real-space movement. The scattering intensities from all rotations were then mapped in reciprocal space. An image of the total intensity of all angles is shown in Fig. 6 ▸(*b*). The real-space variation of the position of the Bragg peak in reciprocal space, **q**_B_(*y*, *z*), can reveal minute shifts due to strain and bending (Etzelstorfer *et al.*, 2014 ▸). We chose the measured **q**_B_ in the middle of the nanowire as reference for a fixed orthogonal reciprocal-space coordinate system. The coordinate system, shown in Fig. 2 ▸, has *q*_3_ nominally parallel with the nanowire axis, *q*_2_ parallel with the membrane and orthogonal to the nanowire, and *q*_1_ orthogonal to both nanowire and membrane. Thus, *q*_3_ is the main coordinate, |**q**_B_| ≃ *q*_3_, while the orthogonal directions *q*_1_ and *q*_2_ relate to tilting of the crystal lattice. Note that *q*_1_ and *q*_2_ cannot be directly related to any crystal direction. At the reference point in the middle of the nanowire, *q*_1_ = *q*_2_ = 0 and |**q**_B_| = *q*_3_. Gaussian peak fits were carried out to quantify the coordinates of **q**_B_. The COM was also evaluated, but we found peak fitting to be more resilient to background noise. Outside of the high-intensity region, the peak fits of the *q*_1_ component were not sufficiently reliable to analyze further. This mapping and peak fitting was repeated for each real-space point, giving two-dimensional images of the *q* positions (Figs. 6 ▸ *c* and 6 ▸ *d*).

The length of **q**_B_, |*q*_B_|, which is related to the (111) lattice plane distance, was 1.832 Å^−1^, which is slightly less than the calculated 1.85 Å^−1^. The deviation of about 1% is probably because the detector distance was not thoroughly calibrated, since we are concerned with relative variations within the nanowire. The relative variation in |**q**_B_| along the nanowire axis was only of the order of 10^−4^. The *q*_2_ component, however, showed a clear gradient along the nanowire axis, which suggests that the lattice was rotated around *q*_1_. From the shift in *q*_2_, the rotation around *q*_1_ can be calculated for every spatial point as β = arctan(−*q*_2_/*q*_3_) ≃ −*q*_2_/*q*_3_. The rotation was averaged perpendicularly to the nanowire axis and plotted *versus* the real-space coordinate along the nanowire, *s* (Fig. 5 ▸ *c*). For most of the range, there is a linear dependence which can be fitted with a gradient dβ/d*s* = −1.78 mrad µm^−1^. Therefore, the orientation in this region can be described by a single radius of curvature of *R* = |d*s*/dβ| = 0.56 mm. The real-space deflection from an ideally straight nanowire in this 1 µm segment is only about 2 nm, which is too small to observe directly.

## 6. Discussion   

The small-angle scattering images showed a relatively homogeneous nanowire, with a thicker head region. To be able to distinguish between the different layers in the core–shell structure the real-space resolution should be improved to about 10 nm. The SBM images showed two strongly scattering regions, positioned roughly at the base and the middle of the nanowire. The Bragg signal at the top part of the nanowire was very weak in the angular range investigated here. The reason for this inhomogeneity is unclear, but two reasons can be mentioned.

First, InP nanowires typically grow in a mix of cubic zincblende and hexagonal wurtzite crystal structure, which differ in the lattice plane spacing and therefore Bragg angle (Kriegner *et al.*, 2011 ▸). We were nominally measuring the Bragg reflection for the zincblende (111) planes, whose scattering vector is only 0.36% larger than that of the wurtzite 000.2 reflection (Kriegner *et al.*, 2011 ▸). The crystal structure depends on many parameters, of which doping is the most relevant here (Wallentin & Borgström, 2011 ▸). The p-i-n doping profile leads to regions with different proportions of wurtzite and zincblende, with more zincblende at the base and less at the top, which in turn leads to variations in the lattice parameter.

Second, the ITO and SiO_2_ films stress the nanowire core, and since the tip has a much thicker ITO layer, the strain could be so strong that the Bragg scattering at the tip was out of the investigated angular range. The SBM method is time consuming, which unfortunately prevented us from making scans at a large range of angles. Weak Bragg scattering may also be due to broadening caused by gradients in the lattice parameter, axially or radially, since the signal is a coherent superposition of scattering from many lattice planes. The top part of the processed nanowire is characterized by steep changes in layer thicknesses, and possibly strong strain gradients.

The SBM measurements also revealed that the nanowire was bent in the plane of the membrane. We tentatively attribute this to an asymmetric thickness of the ITO film. In the scanning electron microscopy image in Fig. 1 ▸(*c*) and in the ptychographic image in Fig. 3 ▸(*f*), the head of the nanowire is slightly thicker on the lower side. This asymmetry is a geometric effect of the ITO sputtering. The side of the nanowire facing the center of the sample, and therefore the ITO source, was coated with a little more ITO, while the other side was slightly shadowed. The uneven thickness could give an asymmetric stress, which in the flexible nanowires can lead to bending. Note that the asymmetric ITO thickness, or even the orientation of the head region, could not have been identified only from the SBM images.

## 7. Conclusions   

In conclusion, we have demonstrated that small-angle scattering can be used for aligning and tracking the real-space position of a nanowire with high resolution. The alignment method used here is deterministic, in the sense that a particular object can be selected and then rotated to the correct Bragg angle. Crucial for the reciprocal-space alignment in our experiment was the preexisting knowledge that the (111) planes are orthogonal to the nanowire axis. However, it is a general property of crystals to exhibit low-energy low-index facets, which means that alignment using facets could be applied to many types of nanocrystals. The measurements also demonstrate how small-angle scattering and Bragg diffraction can give complementary information about a sample, especially when it contains both crystalline and noncrystalline regions.

There are further opportunities with simultaneously collecting the Bragg and small-angle scattering which have not been explored here. In crystalline heterostructures it can be difficult to separate the effects of composition and strain without sophisticated models, since they both affect the lattice constant (Mocuta *et al.*, 2008 ▸; Keplinger *et al.*, 2009 ▸). Since different materials in a heterostructure have different electron density, forward scattering could be used to independently measure the composition. Another possibility is to use the reconstructed probe from small-angle scattering to improve the Bragg imaging. In recent years superresolution methods have been developed for Bragg scattering, based on solving the phase problem by iterative methods similarly to small-angle scattering (Robinson *et al.*, 2001 ▸; Godard *et al.*, 2011 ▸; Hruszkewycz *et al.*, 2012 ▸; Minkevich *et al.*, 2014 ▸). In these coherent Bragg methods, the Bragg peak is a convolution of the sample properties, like lattice tilt, lattice strain and sample shape, with the properties of the probe (Hruszkewycz *et al.*, 2012 ▸; Godard *et al.*, 2011 ▸). Characterizing the probe from the small-angle scattering data could decouple the probe and sample effects, and improve the reconstructions of the Bragg scattering data.

## Appendix A Modified Bragg condition

The nanowires grow in the (111)B direction, and we use the Bragg reflection from the (111) planes which are orthogonal to the growth axis. The direction of the nanowire, in the real-space coordinate system

is

To fulfill the Bragg condition, we rotate the sample around the *z* axis by the angle . The direction of the rotated nanowire is then

For the (111) planes, the reciprocal lattice vector **G** with length 2π/*d* is parallel to the nanowire axis. Thus,

The incident wavevector of the primary beam is

The Bragg condition can be written as

Since the scattered wavevector , this can also be used to calculate the direction of the Bragg reflection and the expected position at the detector.
